# A study on the impact of mental health problems on the academic buoyancy of medical students and the mechanisms

**DOI:** 10.3389/fpubh.2023.1229343

**Published:** 2024-01-16

**Authors:** Bin Hu, Yue Wang, Hai Tao Zhou, Min Li, Li Zheng

**Affiliations:** School of Public Health, Xuzhou Medical University, Xuzhou City, Xuzhou, China

**Keywords:** academic buoyancy, mental health problems, anxiety, influencing factors, psychological

## Abstract

**Objective:**

To analyze the effects of different family environmental backgrounds and mental health problems on academic buoyancy and to explore the potential mechanisms of their effects, using a sample of 2085 medical students in Jiangsu province.

**Methods:**

Using the multiple linear regression to analyze the impact of mental health problems on academic buoyancy in different family environment contexts.

**Results:**

(1) Higher family income and parental literacy implied higher levels of academic buoyancy in children; (2) mental health problems and academic buoyancy were negatively related, and generalized anxiety and uncertainty stress negatively predicted academic buoyancy levels; (3) uncertainty stress may have an indirect effect on academic buoyancy levels through a partially mediating effect of generalized anxiety.

## 1 Introduction

The concept of academic buoyancy was first introduced by Martin and Marsh’s team in 2008, who defined ‘academic buoyancy’ as a student’s ability to deal with the frustrations and challenges of school on a daily basis (1). “Academic buoyancy” is also referred to by Martin’s team as “everyday academic resilience” or “everyday academic resilience,” the nomenclature of which is derived from another concept in psychology, “academic resilience,” which refers to the ability of students to adapt to significant negative academic events (2). But for most students, academic difficulties are rarely long-term and significant (3). Martin and Marsh argue that students actually experience temporary difficulties and setbacks in their daily academic lives more frequently than major setbacks, such as failing a final exam or being criticized by a teacher in a particular class. As a result, the idea of ‘everyday academic resilience’, which focuses more on the temporary difficulties, frustrations and challenges that arise in everyday academic life, has been proposed. At the same time, similar theories have been proposed by national researchers (4).

Martin states that academic buoyancy can help students to adapt to the unexpected events in their daily lives and thus achieve a better state of learning (5). National researchers have found that academic buoyancy is not only a direct predictor of student achievement, but also correlates with student life satisfaction and positive levels of psychological well-being (6–8). Academic buoyancy also affects students’ engagement in learning, which is a key area of research in education. Engagement in learning refers to the extent to which students are emotionally, cognitively and behaviorally engaged in the learning process (9) and is one of the most important indicators of the quality of learning (10). It can affect not only a learner’s current academic performance (11), but also their future progression to higher education and work (12). Specifically, the higher the ability of individuals with high academic buoyancy to cope with difficulties in learning, the higher their evaluation of themselves and their learning, and the higher their satisfaction.

Theories and research on academic buoyancy have revealed that factors affecting academic buoyancy include individual psychological factors, family and environmental factors (13). Among the effects of parenting style and home environment on academic buoyancy, both parenting style and daily communication can influence students’ daily academic performance (14). A positive parenting style enables students to have a better ability to cope with academic setbacks in their normal studies (15). As the primary living environment for students, differences in the home environment can also determine different levels of academic buoyancy. Everyday interpersonal relationships also affect academic buoyancy, which can be enhanced by strengthening relationships between teachers and students. In other words, academic buoyancy is significantly and positively related to teacher-student relationships (16). From an individual psychological perspective, positive psychological factors in individuals can lead to better levels of academic buoyancy (17).

A previous study showed that students’ self-impediments negatively predicted levels of academic buoyancy, examining the factors influencing academic buoyancy from an adverse psychological perspective (10). However, research on mental health issues in relation to academic buoyancy levels is still at an early stage. Mental health issues accompany everyone in their daily studies or lives, and being a medical student often comes with greater academic stress, which can lead to more serious mental health problems (18). The most typical emotions include depression, anxiety and uncertainty and stress. Depression is one of the most common mental health problems among university students (19). The World Health Organization has suggested that a quarter of all university students in China have experienced depression to some degree. Depression is something that everyone experiences, but without proper management and guidance it can turn into depression, which can lead to more uncontrollable and serious consequences. Nowadays, depression has become the “invisible killer” of university students (20). Similar to depression, generalized anxiety is a psychological problem that receives a lot of attention. University students are at the interface of school and society and usually encounter multiple stressors, which are the most important source of anxiety (21). Various pressures such as daily academic stress, stress about future employment and mental health stress in daily life of university students can all add to the level of anxiety, with medical students once again being that more stressed group (22).

Uncertainty stress and social anxiety are new areas that have been gaining attention in recent years and have been studied in numerous other fields such as psychology, public health and clinical medicine (23). Uncertainty stress is a subjective feeling and experience brought about by uncertainty. Social anxiety refers to the fact that university students are in a new environment and need to face different people and things. For some university students who are introverted or have less social experience, this may make it difficult and stressful for them, resulting in social anxiety (24).

The subjects of this study were all from medical schools. Past research has shown that medical students tend to have more severe negative psychological emotional problems due to the special educational system (25). This paper explores the factors that influence academic buoyancy and proposes practical and realistic measures at the school level and the individual student level to improve the overall level of academic buoyancy of students. Finally, the positive impact of academic buoyancy on students is used to help them in their daily lives and achievements. In this experiment we examined family environmental factors and several representative and widespread mental health problems (depression, generalized anxiety, uncertainty stress and social anxiety). The association between these factors and academic buoyancy and the impact on academic buoyancy is examined and the potential mechanisms of action are further explored. Regarding academic buoyancy, previous studies at home and abroad have pointed out that it is the ability of students to self-regulate and relieve themselves in the face of difficulties and stress in their academic life. Students with higher levels of academic buoyancy can better cope with difficulties and better complete their studies. Previous studies have paid less attention to the impact of adolescent psycho-behavioral problems on academic buoyancy, and this study focuses on this.

## 2 Methodology

### 2.1 Objects

A questionnaire survey of undergraduate students enrolled in a medical university in the eastern region of China was conducted in November–December 2022. Stratified random sampling was used. Firstly, the study population was stratified by grade level. Secondly, a number of classes were selected proportionally within the stratum according to the number of students in different majors. Finally, all students within the class were used as respondents. A total of 2,273 questionnaires were distributed and 2085 valid questionnaires were returned, with an effective rate of 91.7%. The survey was approved by the Ethics Committee of Xuzhou Medical University with the informed consent of the participants (approval number: XYFY2020-KL219-01). Due to the limitation of the questionnaire content, we can only collect to get the basic information of family environment factors. The questionnaires collected in this experiment were all in the same university, so the most of the students live in a relatively concentrated area with a relatively stable regional economic level, which allows us to place the research perspective more under the family environment context. The questionnaire was administered through a stratified random sample of different grades and classes in different colleges of the school, and after the classes were selected, the questionnaire was distributed through the counselor of the classes. The initial page of the questionnaire prompts a voluntary option to complete this questionnaire. The sample size for this experiment was calculated to meet the minimum requirements of the design.

### 2.2 General demographic information

Includes gender, age, grade, whether only child, place of residence, monthly household income, type of household, and parents’ education level.

### 2.3 Methods

General demographic information: gender, age, grade, whether the child is an only child, place of residence, monthly household income, type of household, parents’ educational level. Academic Float Scale: This scale was developed by Martin and Marsh (1).

A revised version of Sun Wei wen’s Chinese version of the Academic Float Scale, with four entries in one dimension, was used to assess students’ ability to successfully deal with everyday academic setbacks, difficulties and challenges.

The Likert 5-point scale was used for scoring, with higher scores indicating higher levels of academic buoyancy for the subjects. The Cronbach coefficient for this scale in this study was 0.920.

Uncertainty Stress Scale: This scale consists of 10 entries measuring uncertainty about life, social change, goals and social values in the last 2 weeks. The scale was scored using the Likert 5-point scale, with the total score being the cumulative score for each entry, the higher the score, the greater the uncertain psychological stress. The Cronbach alpha coefficient for this scale in this study was 0. 949.

The Patient Health Questionnaire-9 (PHQ-9 scale): Based on the nine criteria for depression in the Diagnostic and Statistical Manual of Mental Disorders published by the American Psychiatric Association, this questionnaire assesses an individual’s level of depression over the past 2 weeks. There are 9 items in total, and individual items are scored using a scale of 0 (not at all) to 3 (almost every day). Higher scores on the scale indicate more severe depressive symptoms in the respondent. The Cronbach alpha coefficient for this scale in this study was 0.921.

Generalized Anxiety Disorder (GAD-7 scale): This questionnaire assesses an individual’s level of anxiety over the past 2 weeks and consists of seven items, with individual items scored on a scale of 0 (not at all) to 3 (almost every day). Higher scores on the scale indicate more severe anxiety symptoms in the respondent. The Cronbach alpha coefficient for this scale in this study was 0.948.

Social Anxiety Subscale of the Self-Consciousness Scale (SASSC): This scale contains six items, each scored on a scale of 0 (not at all) to 3 (very much), with the fourth item being scored in reverse. Higher scores on the scale indicate more severe social anxiety. The Cronbach alpha coefficient for this scale in this study was 0.818.

### 2.4 Statistical analysis

Descriptive statistics and correlation analyses were performed using SPSS 26.0. Count data were described by n. Comparisons between groups were made using independent samples t-test or ANOVA, and correlation analyses were performed using Pearson correlation analysis with a two-sided test level of *α* = 0.05. The mediation model was constructed using Model 4 in SPSS Process 4.1 prepared by Hayes, and the significance level of the mediation effect was tested using the Bootstrap method with 5,000 replicate samples.

## 3 Results

### 3.1 Common method deviation test

Because all data collection for this study was in the form of self-reporting, common method bias should be avoided. The study design and measurement process began with procedural controls that emphasized the anonymity of responses and reverse scoring. On the basis of this, statistical control is applied to detect them. On this basis, statistical control is applied for detection. Exploratory factor analysis was conducted on the full range of measures by Harman’s one-way test and the results of these unselected factor analyses indicated that a total of six factors have eigenvalues greater than 1. The variance explained by the first common factor analyzed is 38.15%, which is less than the 40% threshold. There were no cases where only one factor was extracted or where a factor appeared to have particularly high explanatory power. Therefore, there is no serious common methodological bias in this study and the relationship between the variables is plausible.

### 3.2 Descriptive statistics and correlation analysis between variables

Previous studies have shown that background factors in the home environment have a direct impact on a person’s level of academic buoyancy. The results of this study, following a univariate analysis of these family environmental factors, are shown in Table 1. Students from one-child families have significantly higher academic buoyancy scores than students from non-one-child families. Students from urban households had higher academic buoyancy scores than those from rural households. Different household income profiles also lead to significant differences in academic buoyancy scores, with higher household income implying higher academic buoyancy scores. Parental literacy was also significantly associated with academic scores. Table 1 shows the one-way analysis. By testing whether there is a difference in the level of academic buoyancy of the population in the case of different demographic information. Descriptive statistics were performed and control variables were selected.

**Table 1:** Univariate analysis of different sociodemographic characteristics and academic buoyancy scores of university students.

Features	*N*	Average academic buoyancy score	SD	t/F	*P*
Age	2085	14.099	3.483		0.425
Gender				1.655	0.098
Male	869	14.249	3.657		
Female	1,216	13.993	3.349		
One child				2.226	0.026
Yes	1,060	14.266	3.576		
No	1,025	13.927	3.376		
Sexual orientation				2.186	0.088
Heterosexual	1712	14.169	3.447		
Homosexuality	29	13.966	3.591		
Bisexual	123	13.358	3.724		
Uncertainty	221	13.986	3.575		
Grade				0.744	0.562
First-year	1,114	14.124	3.467		
Second-year	803	14.153	3.477		
Third-year	104	13.786	3.361		
Fourth-year	59	13.509	3.958		
Fifth-year	5	13.401	4.879		
Residence				3.227	0.001
City	1,214	14.307	3.524		
Rural	871	13.809	3.404		
Monthly household income				6.813	0.001
Under 2000	130	13.785	3.486		
2000–5,000	483	13.569	3.541		
5,001–8,000	535	14.108	3.315		
8,001–15,000	644	14.205	3.485		
15,001 or more	293	14.864	3.539		
Family type				0.921	0.451
Nuclear family	1795	14.151	3.496		
Single parent family	142	13.788	3.412		
Reuniting family	48	13.958	3.632		
Intergenerational family	82	13.549	3.266		
Other	18	14.222	3.282		
Father’s education level				11.746	0.001
Junior High School and below	783	13.651	3.469		
High school or post-secondary	834	14.254	3.328		
University or above	468	14.573	3.689		
Mother’s education level				5.705	0.003
Junior High School and below	949	13.823	3.361		
High school or post-secondary	782	14.286	3.509		
University or above	354	14.427	3.691		

In conducting the correlation analysis between mental health problems and academic buoyancy, the control variables were whether the child was an only child, low household residence, monthly household income and parental literacy. Biased correlation analyses were conducted for the other variables. The correlation matrix for each psychological score is shown in Table 2, with the results of the bivariate correlation analysis without the addition of control variables above the diagonal line and the skewed correlation analysis after the addition of general demographic information as a control variable below the diagonal line. The results indicate that the negative correlation between academic buoyancy and all other scores holds significantly. With no control variables set, academic buoyancy and uncertainty stress, depression scores, anxiety scores, and social anxiety scores all showed significant negative correlations. Setting family residence, whether or not the child was an only child, parents’ education level and monthly family income as control variables, the biased correlation also showed a significant negative correlation between academic buoyancy and the other four adverse psychological conditions. In Table 1, control variables were selected by analyzing whether there were differences in academic buoyancy scores across demographic profiles. In this case, the control variables “being an only child,” “family residence,” “monthly family income” and “parents’ education level” were selected as control variables. They will be used in the correlation analysis and regression models. Table 2 shows the correlation analysis between the four adolescent mental health problems and academic buoyancy. The results show a negative correlation between academic buoyancy and these four mental health problems. Some other variables in the results of the multiple regression analysis are mentioned in Table 2. The results are placed more logically after the correlation analysis in Table 2.

**Table 2:** Correlation matrix between mental health problems and academic buoyancy.

	Academic buoyancy	Uncertainty pressures	Depression	Generalized anxiety	Social anxiety
Academic buoyancy	1	−0.295^**^	−0.270^**^	−0.281^**^	−0.107^**^
Uncertainty pressures	−0.292^**^	1	0.710^**^	0.675^**^	0.384^**^
Depression	−0.261^**^	0.706**	1	0.857^**^	0.317^**^
Generalized anxiety	−0.277^**^	0.671^**^	0.855^**^	1	0.317^**^
Social anxiety	−0.092^**^	0.374^**^	0.301^**^	0.305^**^	1

^**^Significant correlation at *p* < 0.01 level.

### 3.3 Analysis of the impact of different types of negative psychological emotions on academic buoyancy

As shown in Table 3, the results of the multiple linear regressions constructed after including general demographic information as a control variable. Only uncertainty stress and anxiety reached significance as predictors. Table 2 shows the correlations between the level of academic buoyancy and several common adolescent mental health problems that were the focus of this study. Only if the correlation holds is there a subsequent regression model. Table 2 shows a negative correlation between academic buoyancy and all four adolescent health problems of concern. Table 3 shows the results obtained after adding the control variables screened in Tables 1, 2 to the multiple linear regression model. The uncertainty stress and generalized anxiety can be used as predictors of the level of academic buoyancy.

**Table 3:** Multiple linear regression analysis.

Variables	*B-*value	SE	*β*-value	*t*-value	*P*
Constant term	17.416	0.455		38.295	<0.001
Uncertainty pressures	−0.091	0.014	−0.202	−6.576	**<0.001**
Depression	0.004	0.031	0.005	0.113	0.91
Generalized anxiety	−0.129	0.034	−0.155	−3.769	**<0.001**
Social anxiety	0.03	0.024	0.029	1.284	0.199

The bold values in table means that the variable is statistically significant.

Table 3 shows a multiple linear regression analysis with the inclusion of control variables. In the multiple linear regression model, the independent variables were “uncertainty stress,” “depression,” “Generalized anxiety” and “social anxiety.” The dependent variable is “academic buoyancy.” The control variables are “being an only child,” “family residence,” “monthly family income” and “father’s income.” The dependent variable was “academic buoyancy” and the control variables were “being an only child,” “family residence,” “monthly family income,” “father’s educational level” and “mother’s educational level.”

### 3.4 Intermediary model testing

**Figure 1:**
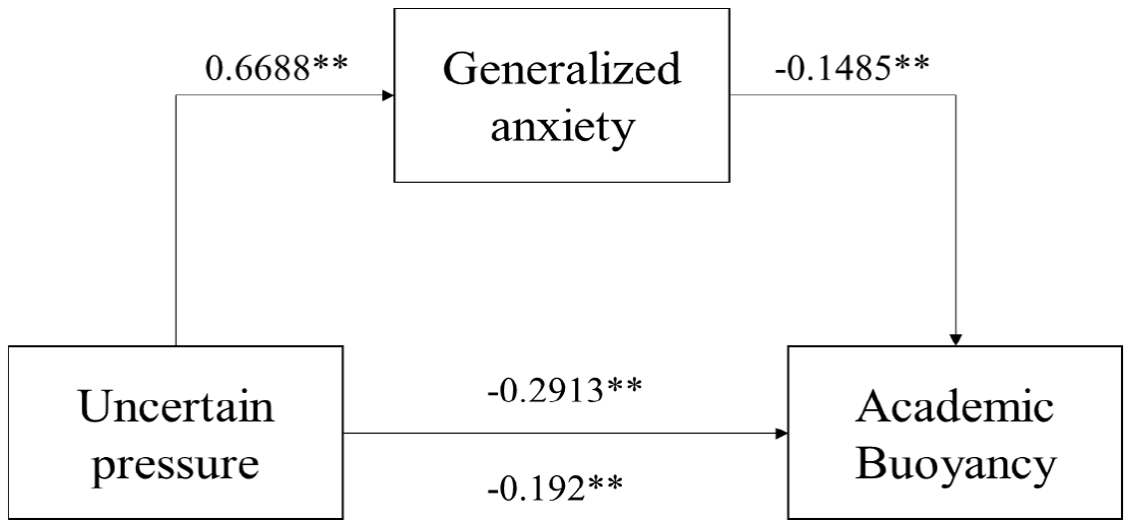
Intermediary model.

As shown in Table 4, the total predictive effect of uncertainty stress on academic buoyancy was significant (standard coefficient = −0.291, *p* < 0.001). After the inclusion of anxiety as a mediating variable, the direct effect still held significantly (standard coefficient = −0.192, *p* < 0.01), as did the indirect effect (standard coefficient = −0.099, *p* < 0.01). At the same time, the Bootstrap 95% confidence interval of the mediated model did not cross 0, indicating that uncertainty stress not only directly predicted academic buoyancy scores, but also predicted academic buoyancy scores through the mediating effect of anxiety. This direct effect was −0.192 and the mediating effect was −0.0993, which accounted for 65.9 and 34.1% of the total effect, respectively, which was a partial mediation. Table 4 shows the mediation effect results. The results indicate the presence of mediating effects. Figure 1 demonstrates the mediating effect. Uncertainty stress besides directly affects the level of academic buoyancy. It also affects the level of generalized anxiety by influencing the level of generalized anxiety. It ultimately affects academic buoyancy.

**Table 4:** The mediating role of generalized anxiety between uncertainty stress and academic buoyancy.

Models	Predictor variables	Result variables	*β*-value	BootLLCI	BootULCI
Model 1	Uncertainty pressures	Academic buoyancy	−0.2913^**^	−0.1132	−0.0591
Model 2	Uncertainty pressures	Generalized anxiety	0.6688^**^	0.3413	0.3816
	Generalized anxiety	Academic buoyancy	−0.1485^**^	−0.1776	−0.0712
	Uncertainty pressures				

## 4 Discussion

This study found that medical students’ academic buoyancy received overall influence from both family environment and personal psychological factors. The level of family income and parental literacy in the home environment significantly affects the child’s level of academic buoyancy, which is similar to the findings obtained from previous studies (14). It is worth noting that this study found that children from non-one-child families had lower levels of academic buoyancy than those from one-child families. The negative correlation between academic buoyancy and all four psychological disorders was significant, and the partial correlation analysis, which controlled for demographic variables, also suggested that the negative correlation was valid, indicating the seriousness of the negative psychological disorders’ negative impact on students’ daily academic life. Higher uncertainty stress and generalized anxiety significantly predicted lower levels of academic buoyancy. These two psychological emotions were the most significant influences in the current study.

According to the Diagnostic and Statistical Manual of Mental Disorders, Fifth Edition (DSM-5), this study classified the anxiety of university students into “social anxiety symptoms,” which is better known as “social phobia” and is diagnosed in psychology as “social anxiety disorder” (26). Both types of anxiety are essentially in the same diagnostic category, but social anxiety has a special significance in the context of this study of medical students. For university students, social stress is suddenly at a peak compared to secondary school and is more likely to have a range of unknown consequences due to positive or negative social influences (27). The two types of anxiety were analyzed as two independent influences in the current study.

The regression model shows that generalized anxiety was the most influential factor on academic buoyancy levels in this experiment. The area of “generalized anxiety” has been a popular area of research for university students, particularly medical students. The cause of generalized anxiety symptoms is not clearly defined and can be a sudden and unexpected event in life, such as health or financial worries, or uncertainty about future career prospects. Generalized anxiety can be defined as a “mental disorder in which prolonged and persistent worry is the central symptom.” Patients worry excessively about life, family, finances, work, school, etc., which are difficult to control and may be accompanied by psychological or physical symptoms (28). This anxiety has many negative consequences for university students, including insomnia, palpitations and nervousness in daily life, inability to concentrate in work and study, frustration and loss of motivation (22). These adverse physical and psychological events can have a direct impact on a person’s level of academic buoyancy. Anxiety can not only affect a student’s academic buoyancy and thus his or her grades and future development, but can also lead to further difficulties in life (29). It is imperative to propose measures to alleviate this situation from the perspective of schools and individuals.

Unlike generalized anxiety, people with high social anxiety have a strong sense of self-focus in their daily lives and when socializing (24). Being very demanding of oneself to the point of distortion and very sensitive to external feedback can lead a person into self-denial and self-doubt (30). This situation not only creates a very bad feeling in the person’s life, but also generates negative feedback for the people around them. Social anxiety is also a risk factor for major depressive disorder (31). Although social anxiety is an abnormal expression, unfortunately it is currently widespread among university students and how to overcome it needs to be thoroughly researched and studied.

Uncertainty stress was another predictor of academic buoyancy levels in this study, and existing research indicates that uncertainty stress is a source of numerous mental health problems. Previous research has suggested that higher levels of uncertainty stress among college student populations may contribute to elevated suicidal ideation (32). In a study of Swedish physicians, uncertainty stress was shown to lead to increased levels of anxiety (33). In the present study, we can also see a positive association between uncertainty stress and generalized anxiety and social anxiety. Uncertainty stress is typically manifested when an individual is faced with an unknown situation in life, work or study, which can lead to negative events. Uncertainty stress in this study led to a decrease in academic buoyancy levels, with a range of negative consequences.

Among the many factors influencing academic buoyancy, uncertainty stress and generalized anxiety at the psychological level of medical students play an important counter-catalytic role. After confirming its negative predictive effect on academic buoyancy, its possible mechanisms of action were further explored. It has been suggested that uncertainty stress will exacerbate levels of anxiety, and after adding general demographics as covariates to the model, the mediating role of uncertainty stress, generalized anxiety and academic buoyancy was analyzed. The results showed that the mediating relationship held and that generalized anxiety played a partially mediating role in the mediating model. This result suggests that the influence of psychological factors on individuals is not only direct, but that the complex effects of mutual mediation and interaction may have more serious consequences. This will require us to add more mental health issues in subsequent studies and continue to explore possible mechanisms in depth.

There are still some parts of the current study that need improvement. Firstly, this study used cross-sectional data with a weak level of causal validation; future research could further explore causality by following up with the university students in this study to obtain longitudinal data. Secondly, all questionnaires in this study are self-administered, which makes it difficult to ensure the quality of the survey information, and those who fill in the questionnaires may have wrong or disorganized answers, which can lead to a reduction in the quality of the survey treatment. Thirdly, there are many mental health problems of medical students that have not been included in the study apart from depression, anxiety and uncertainty stress mentioned in this study. Research could include more emotions such as sadness, anger, fear and hatred, in order to explore the mechanisms influencing medical students’ academic buoyancy in more depth and provide positive insights to improve the level of academic buoyancy among university students.

## 5 Conclusion

1. The home environment has a very strong influence on the level of academic buoyancy of students. Parents with high income and literacy levels mean that children have higher levels of academic buoyancy. The family environment and education remain the most important factors influencing a child’s development. The education and guidance of parents will determine the future development of their children.
2. Negative psychological mood and academic buoyancy levels were significantly and negatively correlated, and the negative prediction of generalized anxiety and uncertainty stress on academic buoyancy held true. Anxiety can be used as a mediating variable to mediate the effect of uncertainty stress on academic buoyancy.

## Data availability statement

The original contributions presented in the study are included in the article/supplementary material, further inquiries can be directed to the corresponding author.

## Ethics statement

The survey was approved by the Ethics Committee of Xuzhou Medical University with the informed consent of the participants (approval number: XYFY2020-KL219-01). The participants provided their written informed consent to participate in this study.

## Author contributions

BH: research idea development, research process coordination, research topic selection, design, and data processing and analysis. YW: writing thesis. HZ: data checking and analysis, and revising the thesis. ML: research supervision, statistical analysis of data, and participation in data analysis and interpretation. LZ: research supervision, statistical analysis of data, and participation in data analysis and interpretation. All authors contributed to the article and approved the submitted version.
